# Supplementary material for: Investigation of Zur-regulated metal transport systems reveals an unexpected role of pyochelin in zinc homeostasis
Source: mBio. 2024 Sep 24;15(10):e02395-24. doi: 10.1128/mbio.02395-24 (PMC11481552; doi:10.1128/mbio.02395-24)
Supplement: Supplemental material — Fig. S1-S9; Tables S1 and S2. [file mbio.02395-24-s0001.docx]

**List of Supplementary Material**

**Figure S1**: Gene expression analyses in the early exponential growth phase.

**Figure S2**: Gene expression analyses of *PA4066* in the *PA4065* mutant strain.

**Figure S3**: Growth comparison of the quadruple mutant *PA4065PA2911znuAzrmB* with parental strains

**Figure S4**: pLDDT values

**Figure S5**: Pigmentation of stationary phase cultures of wild-type PA14 and mutant strains *znuAzrmB*, *PA4065znuAzrmB*, and *PA2914znuAzrmB*, grown in VBMM

**Figure S6**: Positive mode ESI spectra of the supernatants (fraction extracted with ethyl acetate) from *Pseudomonas aeruginosa* PAO1 and PA14.

**Figure S7**: Intracellular Fe, Mn, Ni, and Cu content

**Figure S8**: Intracellular Zn and Co content in PA14 wild type, *PA4065*, *PA2914*, *pchE* and *PA2914pchE* strains

**Figure S9**: Effect of Co supplementation on PA14 wild type and *znuAzrmB* strains.

**Table S1**: Bacterial strains and plasmids used in this work.

**Table S2**: List of primers used in this work.

**Figure S1**

**Gene expression analyses in the early exponential growth phase.** RT-qPCR was performed on bacterial samples grown in E-VBMM. Each sample was collected at the early exponential growth phase (corresponding to approximately 0.3 OD_600_), and RNA was extracted and retrotranscribed for the RT-qPCR reaction. **(A)** Relative fold induction of *PA2911* and *PA4063* in the wild-type (grey bars) and *znuAzrmB* mutant (blue bars) grown in E-VBMM supplemented with ZnSO_4_ 3 mM compared to the gene expression in the wild-type grown in E-VBMM. Statistical analyses were performed by Two-way ANOVA and Bonferroni's multiple comparison test. Asterisks indicate statistically significant differences (*****p* < 0.0001). **(B)** Relative fold induction of *PA2911*, *PA4063*, *ftpA*, *ftpX*, *pchD* and *pchE* in the *znuAzrmB* mutant strain, compared to the wild-type strain. Data are mean values ± S.D. of triplicates, and statistical analysis was performed by Paired T test. Asterisks indicate statistically significant differences (*****p* < 0.0001).

**Figure S2**

**Gene expression analyses of *PA4066* in the *PA4065* mutant strain.** RT-qPCR was performed on PA14 wild-type and PA4065 mutant strain, grown in E-VBMM. Relative fold induction of *PA4066* in the *PA4065* mutant strain was calculated as described in Material and Methods by comparing it to the fold induction in the wild-type strain and using *rpoD* as the housekeeping gene. Data are mean values ± S.D. of triplicates, and statistical analysis was performed by Paired T-test. Asterisks indicate a statistically significant difference (***p* < 0.01).

**Figure S3**

**
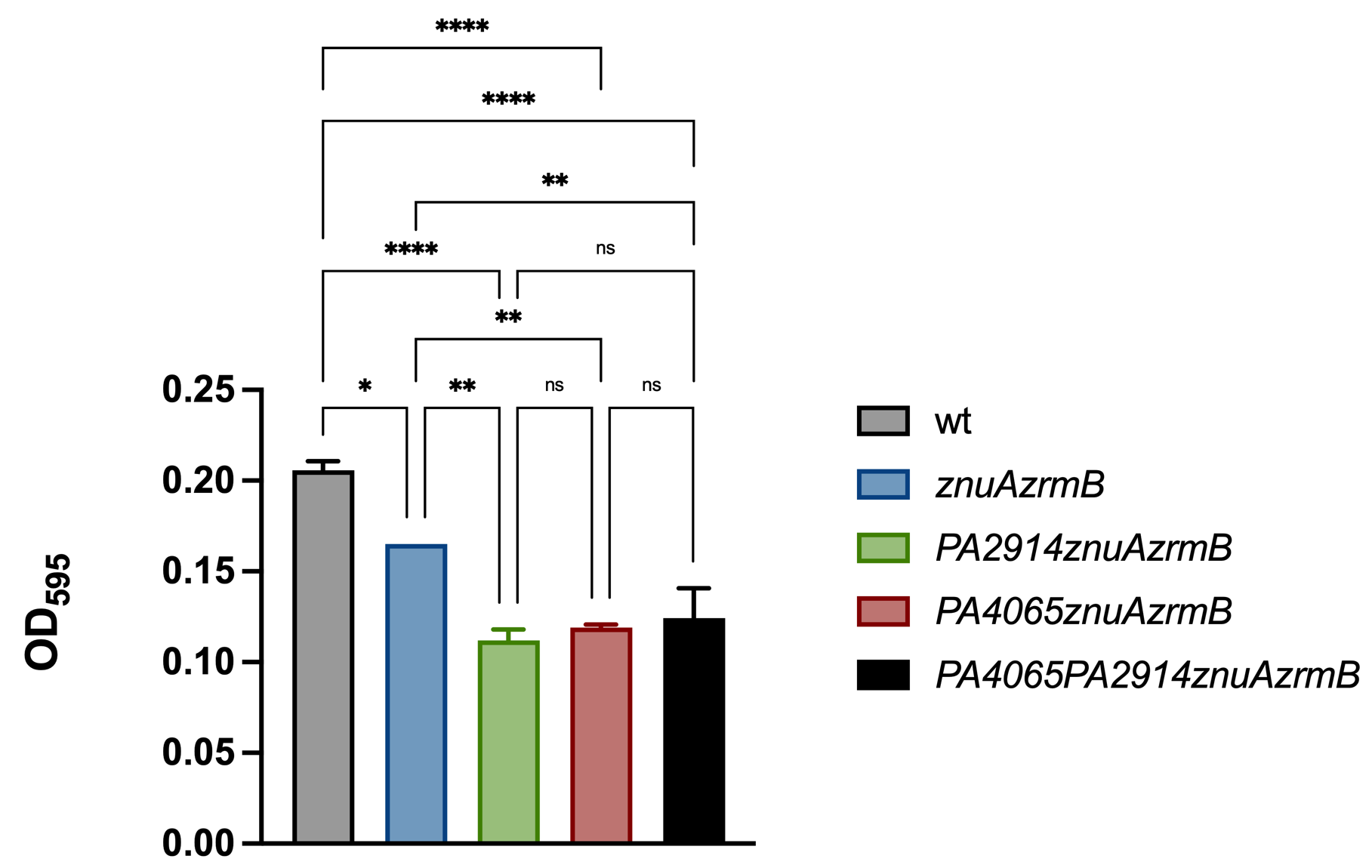
**

**Growth comparison of the quadruple mutant *PA4065PA2911znuAzrmB* with parental strains.** The simultaneous deletion of *PA4065* and *PA2914* in the *znuAzrmB* background does not cause any significant growth difference compared the triple mutants. Bars represent mean values with SD of three independent cultures. Statistical significances were calculated by ordinary one-way ANOVA and Tukey’s multiple comparison test. Asterisks indicate statistically significant differences (**p*<0.05; ***p*<0.01; *****p* < 0.0001); *ns*: not significant)

**Figure S4**

**
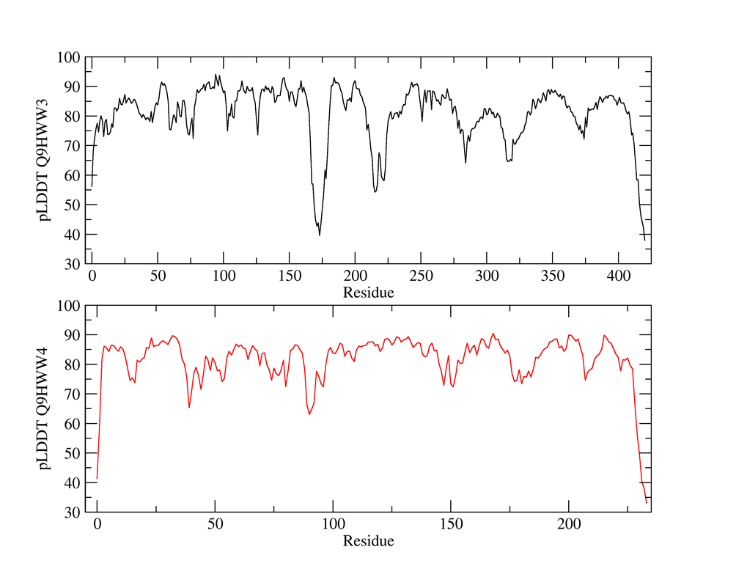
**

**pLDDT values.** Values are obtained as a function of the sequence observed for the structures predicted by Alphafold 2

**Figure S5**

**
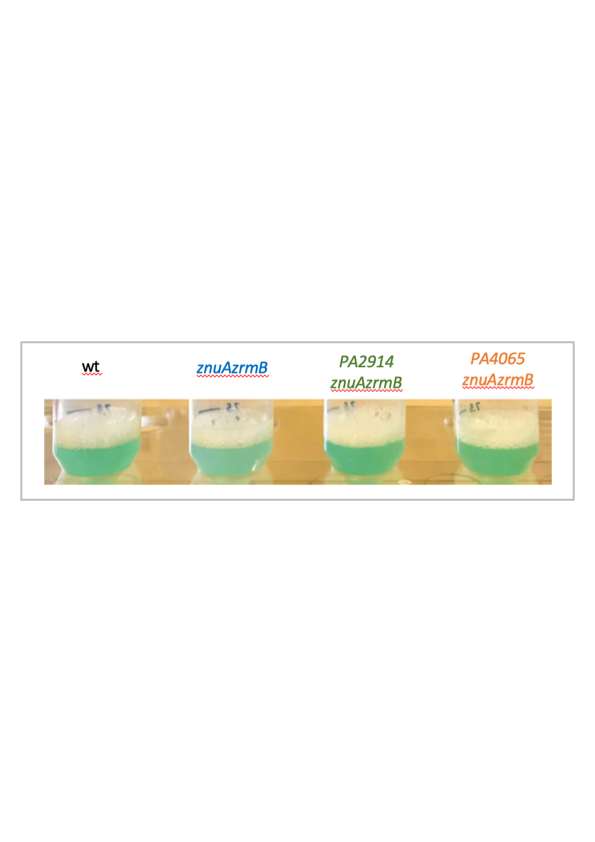
**

**Pigmentation of stationary phase cultures of wild-type PA14 and mutant strains *znuAzrmB*, *PA4065znuAzrmB*, and *PA2914znuAzrmB*, grown in VBMM**. The *znuAzrmB* strain shows a different pigmentation compared to the other strains.

**Figure S6**

1. **PAO1 wild type**


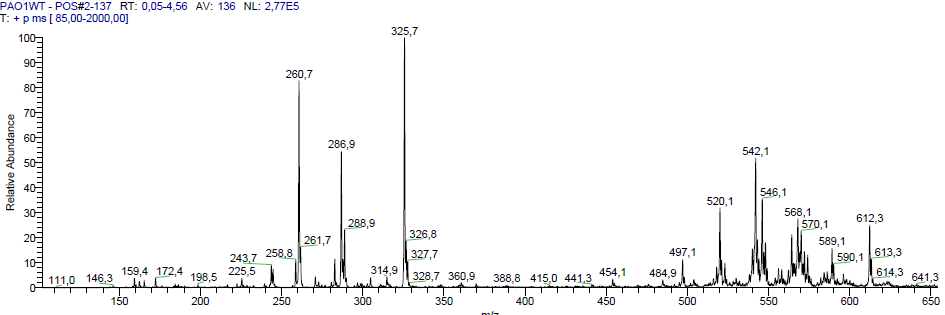


1. **PAO1 *pchD***


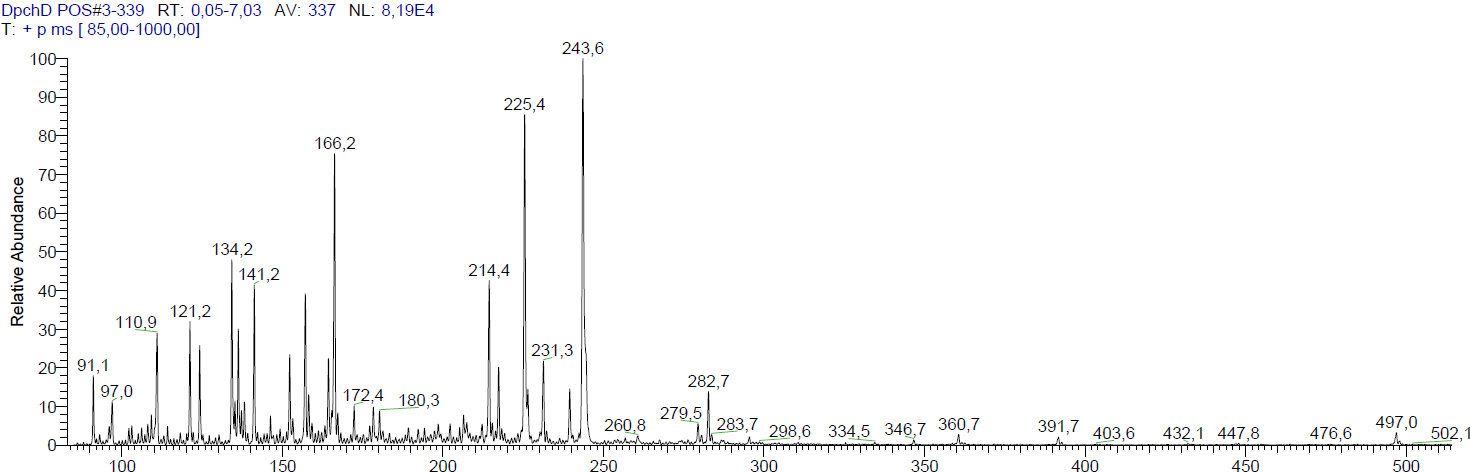


1. **PA14 wild type**


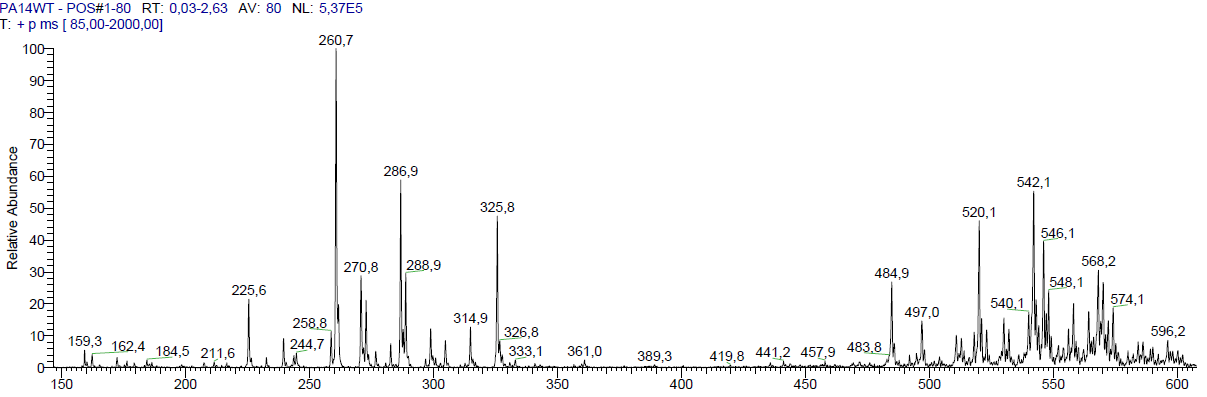


1. **PA14 *znuAzrmB***


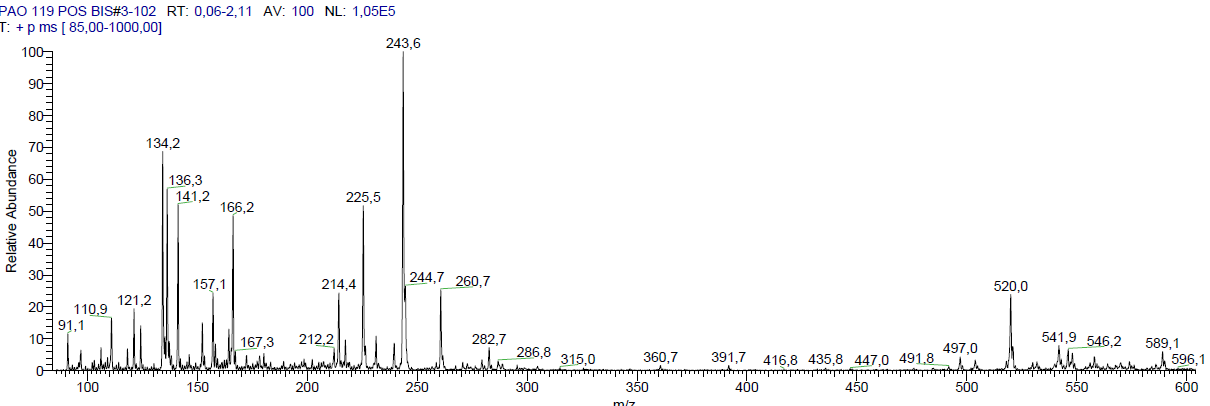


1. **PA14 *PA2914znuAzrmB***


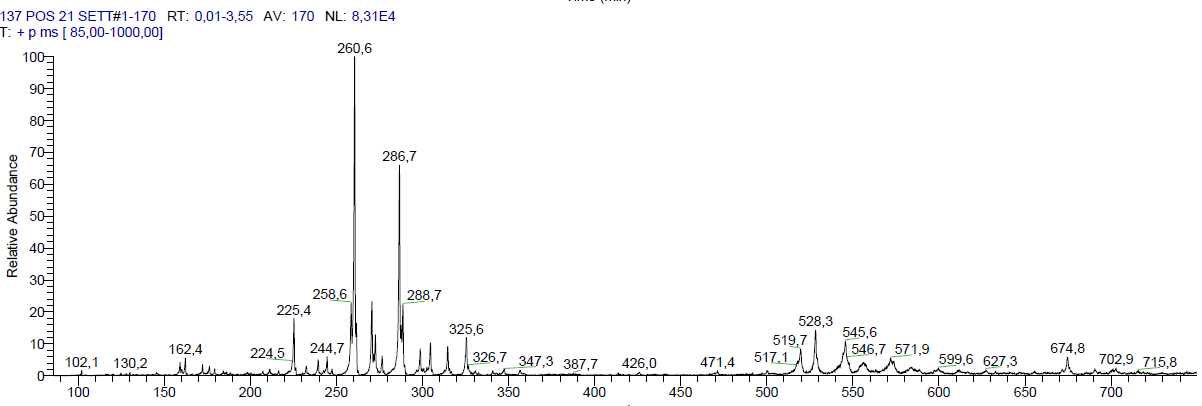


1. **PA14 *PA4065znuAzrmB***


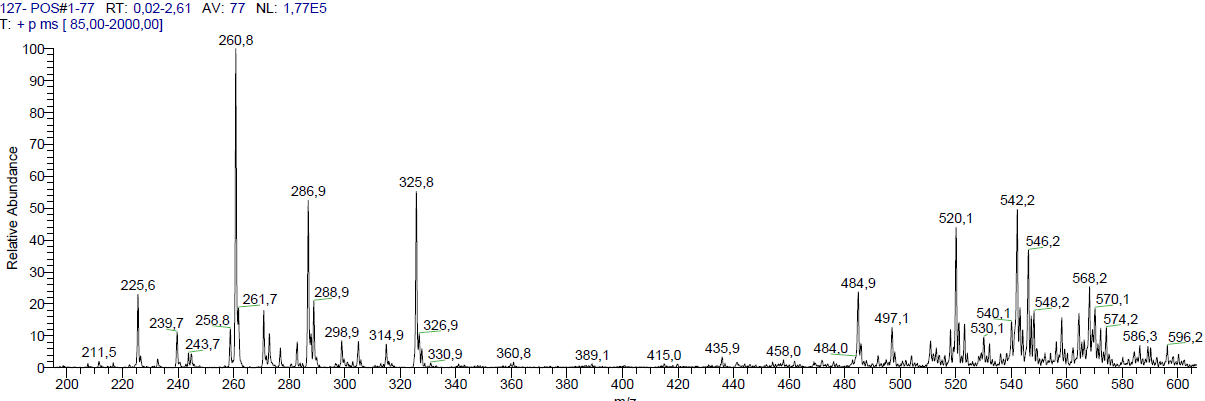


**Positive mode ESI spectra of the supernatants (fraction extracted with ethyl acetate) from *Pseudomonas aeruginosa* PAO1 and PA14.**

The mass spectra were recorded on a ThermoFinnigan LCQ Classic LC/MS/MS ion trap equipped with an ESI source and an injection syringe pump. The fractions of the supernatants extracted with ethyl acetate were diluted 1/10 with MeOH/H_2_O (9:1) and were infused in the electrospray system at a constant flow rate of 10 μL/min^−1 and^ the mass spectra were recorded in positive ion mode (spray voltage 6.5kV, capillary voltage 35V, capillary temperature 200°C). ESI-MS data are given as m/z, with mass expressed in atomic mass units (amu). Panels A and B compare the fractions extracted from the wt strain and the *pchD* strain. The spectrum of the sample from the *pchD* strain shows an extremely simplified profile compared to that of the wild strain. In addition to missing the peak corresponding to PCH (m/z 325), all other major peaks are absent in the mutant, including the one with m/z 260 (PQS), the peaks with m/z 286 and 288, likely corresponding to other 4-hydroxy-2-alkylquinolines (HAQs) (1) and all those with m/z above 500, possibly corresponding to rhamnolipids (2). The peaks observed in the spectrum of the mutant represent molecular species that are very scarcely abundant in the wild-type strain. Similarly, the main ion species present in the PA14 wild type strain supernatant (panel C) are absent in the *znuAzrmB* mutant (panel D), whose composition is similar to that of the *pchD* mutant reported in panel B (absence of peaks for PCH and PQS). In contrast, the mass spectrum of the *PA4065znuAzrmB* mutant (panel F) is very similar to that of the wild-type strain. The peaks corresponding to the various HAQs and PCH are also restored in the *PA2914znuAzrmB* triple mutant, although in this case the peak for PCH is less pronounced than those of HAQs.

**Figure S7**

**Intracellular Fe, Mn, Ni, and Cu content.** ICP-MS analyses of bacteria grown for 20 hours in E-VBMM with trace metals (0.2 μM ZnSO_4_ and 0.1 μM FeSO_4_, NiSO_4_, Co(NO_3_)_2_, CuSO_4_ and MnCl_2_). Bars are the mean value of three biological replicates ± SD. Statistical analyses were performed by One-way ANOVA. Differences with *p* < 0.01 were considered statistically significant. Asterisks indicate statistically significant differences between mutant strains and wild-type (***p* < 0.01; ***p < 0.001; *****p* < 0.0001); hash signs indicate pairwise statistically significant differences (^###^*p* < 0.001).

**Figure S8**

**Intracellular Zn and Co content in PA14 wild type, *PA4065*, *PA2914*, *pchE* and *PA2914pchE* strains.** ICP-MS analyses of bacteria grown for 20 hours in E-VBMM with trace metals (0.2 μM ZnSO_4_ and 0.1 μM FeSO_4_, NiSO_4_, Co(NO_3_)_2_, CuSO_4_ and MnCl_2_). Bars are the mean value of three biological replicates ± SD. Statistical analyses were performed by One-way ANOVA. Asterisks indicate statistically significant differences between PA14 wild-type and all mutant strains (***p* < 0.01; ****p* < 0.001; ****p < 0.0001); hash signs indicate statistically significant differences between *PA4065* and the other mutant strains (^####^*p* < 0.0001).

**Figure S9**

**Effect of Co supplementation on PA14 wild type and *znuAzrmB* strains.** Bacteria were grown in E-VBMM (continuous lines) and E-VBMM + Co(NO_3_)_2_ 0.010 mM (dotted lines). Each symbol indicates the mean ± SD of triplicates, and lines represent nonlinear fit according to the Logistic Growth Equation.

**Table S1: Bacterial strains and plasmids used in this work.**

| ***Strain*** | ***Relevant genotype*** | ***Reference or source*** |
| --- | --- | --- |
| ***E. coli*** |  |  |
| DH5α | φ80 ∆*lac*Z 15∆ (*lac*-*arg*F) U169 *deo*R*rec*A1 *end*A1 *hsd*R17 (rk^-^, mk^+^) *pho*A*sup*E44 λ^-^*thi*-1 *gyr*A96 *rel*A1 | Lab collection |
| HB101 | *F^-^ mcrB mrr* *hsdS20*(r_B_^-^ m_B_^-^) *rec*A13 *leu*B6 *ara*-14 *pro*A2 *lacY1* *gal*K2 xyl-5 mtl-1 *rps*L20(Sm^R^) glnV44 λ^-^ | Lab collection |
| ***P. aeruginosa*** |  |  |
| **PA14** | **wild type** | **Lab collection** |
| *znuA*  (MDO101) | *znuA::Gm* | (3) |
| *znuAzrmB*  (VS-MDO119) | *znuA::scar zrmB::Gm* | This study |
| *PA2914*  (VS-MDO129) | *PA2914::Gm* | This study |
| *PA2914znuAzrmB*  (VS-MDO137) | *PA2914::scar znuA::scar zrmB::Gm* | This study |
| *PA4065*  (MDO118) | *PA4065::Gm* | This study |
| *PA4065znuAzrmB*  (VS-MDO127) | *PA4065::scar znuA::scar zrmB::Gm* | This study |
| *PA4065PA2914znuAzrmB*  (VS-MDO152) | *PA4065::scar znuA::scar zrmB::scar PA2914::Gm* | This study |
| *pchE*  (VS-MDO153) | *pchE::Gm* | This study |
| *znuAzrmBpchE*  (VS-MDO154) | *znuA::scar zrmB::scar pchE::Gm* | This study |
| *PA2914znuAzrmBpchE*  (VS-MDO155) | *znuA::scar zrmB::scar PA2914::scar pchE::Gm* | This study |
| *PA4065znuAzrmBpchE*  (VS-MDO156) | *znuA::scar zrmB::scar PA4065::scar pchE::Gm* | This study |
| *PA2914pchE*  (EM-MDO218) | *PA2914::scar pchE:Gm* | This study |
| **PAO1** | **wild type** | **Lab collection** |
|  | *ΔpchD* | Lab collection |
|  | *ΔpvdAΔpchDΔfpvA PpchE::lux* | (4) |
|  | *ΔpvdA* | (5) |
| ***Plasmids*** | ***Description*** | ***Reference*** |
| pEX18Tc | Broad-host-range gene replacement vector with MCS from pUC18; *sacB*+;TcR,*oriT*+ | (6) |
| pRK2013 | Broad-host-range helper vector; *ColE1*-Tra(RK2)+,kanR | (7) |
| pFLP2 | BhrFlp recombinase-producing plasmid | (6) |
| p*zrmA*PTZ110 | Reporter plasmid bearing a *zrmA* promoter::*lacZ* fusion | (8) |
| pPS856 | Source of gentamicin resistance cassette. AmpR, GmR | (6) |

**Table S2: List of primers used in this work.**

| **Primers for mutant strains construction:** | |  |
| --- | --- | --- |
| **Primer** | **Sequence (5'-3')** | **Description** |
| PA2914_1 | ATAGAATTCGTGTTCCTCTACGACAGC | Forward primer to amplify the 5' region of *PA2914* |
| PA2914_2 | ATAGGATCCGATCAGCCAGACGATGTG | Reverse primer to amplify the 5' region of *PA914* |
| PA2914_3 | ATAGGATCCCTGTGGGTCTTCATCTGC | Forward primer to amplify the 3' region of *PA2914* |
| PA2914_4 | ATAAAGCTTTGTTCATGCCCGACTACG | Reverse primer to amplify the 3' region of *PA2914* |
| PA2914_5 | TCATCGTCATCGTCGACTAC | Forward primer to check *PA2914* deletion |
| PA2914_6 | TTCATGTGCCACGACTACAC | Reverse primer to check *PA2914* deletion |
| PA4065_5 | CTCGAATTCGGCGTTTCTCCAGTTGCTCT | Forward primer to amplify the 5' region of *PA4065* |
| PA4065_6 | CGCGGATCCCTTGTCGTCGTGCTTGACCA | Reverse primer to amplify the 5' region of *PA4065* |
| PA4065_7 | CGCGGATCCCAGGCCAACTACGGCATCTA | Forward primer to amplify the 3' region of *PA4065* |
| PA4065_8 | CCCAAGCTTCTGGCGTTCTCCACCTTGAA | Reverse primer to amplify the 3' region of *PA4065* |
| PA4226_9 | CCCGAGCTCATCTAACGAAACAGTCCG | Forward primer to amplify the 5' region of *pchE* |
| PA4226_2 | ATAGGATCCAACATCGGGTGGCGTTGA | Reverse primer to amplify the 5' region of *pchE* |
| PA4226_3 | ATAGGATCCGACCGGGTGATCAGCTTC | Forward primer to amplify the 3' region of *pchE* |
| PA4226_4 | CTCAAGCTTCTCCAGTTGGGTTTCCTC | Reverse primer to amplify the 3' region of *pchE* |
| PA4226_7 | ATCCGCCTGATGTACCTGCA | Forward primer to check *pchE* deletion |
| PA4226_8 | ATCGACCTTGCCATTGCC | Reverse primer to check *pchE* deletion |
| **Primers for RT-qPCR:** | |  |
| **Target gene** | **Forward (5’-3’)** | **Reverse (5’-3’)** |
| *fptA* | ACCTACGAAACCGGGATCAA | GGTCTTCCTGCGGATTGTTC |
| *fptX* | CTGGGTGGTCAAGTTCCTCT | ATCGGCAGGATCCAGCTAC |
| *oprI* | ATTCTCTGCTCTGGCTCTGG | CGGTCTGCTGAGCTTTCTG |
| *PA2911* | CTACATCGACCCCTGGCATC | GCGGTCGATATGGTTCTGGT |
| *PA4063* | CACAAGGAGAAGGCAGGCC | CTGAATTTTCTGGGTGGCGG |
| *pchD* | CCTTCGTCGAGACCTGCTT | CTGATCTCATGCTGGCGATG |
| *pchE* | GATCAATACCATCGACGCGC | GATAGATCGAAGTCCAGCGC |
| *pchR* | CATCACCATCATTGCTCCGC | GGTCACCAGCTTCATATTCGG |
| *PA4066* | TCCTGGTCCCCTACTACGG | GGTACAACTCGTCCATCTGC |
| *rpoD* | CATCGCCAAGAAGTACACCA | CCACGACGGTATTCGAACTT |

**References**

1. Lépine F, Milot S, Déziel E, He J, Rahme LG. Electrospray/mass spectrometric identification and analysis of 4-hydroxy-2-alkylquinolines (HAQs) produced by Pseudomonas aeruginosa. J Am Soc Mass Spectrom. 2004;15(6):862–9.

2. Sharma R, Singh J, Verma N. Optimization of rhamnolipid production from Pseudomonas aeruginosa PBS towards application for microbial enhanced oil recovery. 3 Biotech. 2018;8(1).

3. D’Orazio M, Mastropasqua MC, Cerasi M, Pacello F, Consalvo A, Chirullo B, et al. The capability of Pseudomonas aeruginosa to recruit zinc under conditions of limited metal availability is affected by inactivation of the ZnuABC transporter. Metallomics. 2015;7(6):1023–35.

4. Visaggio D, Pirolo M, Frangipani E, Lucidi M, Sorrentino R, Mitidieri E, et al. A Highly Sensitive Luminescent Biosensor for the Microvolumetric Detection of the Pseudomonas aeruginosa Siderophore Pyochelin. ACS Sensors. 2021;6:3273–83.

5. Imperi F, Putignani L, Tiburzi F, Ambrosi C, Cipollone R, Ascenzi P, et al. Membrane-association determinants of the ω-amino acid monooxygenase PvdA, a pyoverdine biosynthetic enzyme from Pseudomonas aeruginosa. Microbiology. 2008;154(9):2804–13.

6. Hoang TT, Karkhoff-Schweizer RR, Kutchma AJ, Schweizer HP. A broad-host-range Flp-FRT recombination system for site-specific excision of chromosomally-located DNA sequences: application for isolation of unmarked Pseudomonas aeruginosa mutants. Gene. 1998;212:77–86.

7. Figurski DH, Helinski DR. Replication of an origin-containing derivative of plasmid RK2 dependent on a plasmid function provided in trans. Proc Natl Acad Sci. 1979;76:1645–52.

8. Mastropasqua MC, D’Orazio M, Cerasi M, Pacello F, Gismondi A, Canini A, et al. Growth of Pseudomonas aeruginosa in zinc poor environments is promoted by a nicotianamine-related metallophore. Mol Microbiol. 2017;106(4):543–61.
